# Effect of Massage on Stress Indicators in Recreational Horses—A Pilot Study

**DOI:** 10.3390/ani15060789

**Published:** 2025-03-11

**Authors:** Ewa Jastrzębska, Aleksandra Górecka-Bruzda, Magdalena Ogłuszka, Maryla Sylwia Lipka, Aleksandra Pawłowska

**Affiliations:** 1 Department of Horse Breeding and Riding, University of Warmia and Mazury in Olsztyn, Oczapowskiego 5, 10-719 Olsztyn, Poland; 2 Department of Animal Behaviour and Welfare, Polish Academy of Sciences, Institute of Genetics and Animal Biotechnology, Postępu 36A, Jastrzębiec, 05-552 Magdalenka, Poland; a.gorecka@igbzpan.pl (A.G.-B.); m.ogluszka@igbzpan.pl (M.O.)

**Keywords:** welfare, massage, horses, cortisol, stress, conflict behavior, heart rate

## Abstract

This pilot study investigated the effects of massage therapy on stress indicators in recreational horses, focusing on salivary cortisol levels, heart rate and conflict behaviors. Five recreational horses were analyzed under two experimental conditions: without and with massage treatments before riding sessions. Saliva samples were collected at four specific intervals in each condition to measure cortisol concentrations using an ELISA test. Observations of behavioral indicators and heart-rate monitoring complemented the physiological data. The results revealed that massage significantly reduced cortisol levels in saliva, with decreases averaging 526.6 pg/mL and 321.8 pg/mL during key sampling intervals. Behavioral analysis showed a notable reduction in conflict behaviors such as tail swishing, which decreased from 121 occurrences to 85 after massage. Relaxation behaviors, including relaxed lower lip and half-closed eyes, were frequently observed during massage sessions, further confirming its calming effects. These findings demonstrate that massage effectively alleviated stress in horses, providing practical benefits for their well-being and usage in recreational activities. The study underscores the potential of incorporating massage therapy into routine equine care to promote relaxation and enhance animal welfare.

## Simple Summary

Horses face various stressors that can impact their health and performance, including physical exertion, competitions, transportation, veterinary procedures and horse–group interactions. Stress in horses is often measured by increased levels of cortisol, the stress hormone, which can lead to health issues. Non-invasive techniques like saliva tests and behavioral observations are becoming popular for measuring stress. Massage therapy has numerous benefits for horses, such as reducing pain, lowering blood pressure and decreasing muscle tension. This study aimed to examine the effects of massage on stress indicators in recreational horses by comparing cortisol levels, heart rate and conflict behavior before and after massage treatments. The results showed that massage significantly reduced stress in horses, evidenced by lower cortisol levels and fewer conflict behaviors. These findings suggest that massage can improve horse well-being and offer practical benefits for horse owners and caregivers, enhancing the relationship between horse and rider while promoting relaxation and comfort for the animal.

## 1. Introduction

Animals are exposed to various stressors [1]. Various circumstances to which horses are regularly exposed have been classified as potential stress triggers based on increased cortisol release. Among them are physical exertion [2], participation in equestrian competitions [3], transportation [1,2,4,5], veterinary procedures [6,7,8] and interactions with new groups of horses [9,10]. Stressful stimuli can lead to a disruption of homeostasis, which directly affects the health, well-being and physical performance of horses [2,11].

In the majority of conducted studies, cortisol levels in the blood were measured. However, methods for analyzing cortisol in horses’ saliva have also been developed [10]. Non-invasive endocrinological examination techniques are becoming increasingly common because they avoid animals’ reactions to repeated venipuncture, which can cause stress [1]. Non-invasive methods of determining stress in horses include recording behavioral indicators, assessing cortisol levels in saliva [12,13], evaluating glucocorticoid metabolites in feces and urine, indirect blood pressure measurements, monitoring heart rate and determining heart rate variability [14,15,16,17].

During brief stress, horses may exhibit changes in behavior [10]. Behaviors indicating stress in horses include nervous tail swishing or raising and shaking the head. Relaxation-related behaviors include lowering the head, licking and chewing, yawning and loosening the tail [5]. Conflict behavior is the response of horses that encounter difficulties in coping with mental or physical discomfort and typically manifests as a form of resistance to cues and/or equipment during handling or training [18].

Massage has a wide range of positive effects. It helps to relieve the body of pain and stress, reduces blood pressure and muscle tension and aids in achieving relaxation [19,20]. Manual massages are used in horses to improve behavior, performance and well-being, as well as to enhance the relationship between the horse and the rider [5].

The objective of this study was to investigate the effects of massage therapy on stress indicators in recreational horses, specifically focusing on salivary cortisol levels, heart rate and conflict behaviors, to assess whether massage can reduce stress and improve welfare in horses used for recreational activities.

## 2. Material and Methods

The study was conducted on a group of five recreational horses maintained in a stable (box) system with several hours of daily access to paddocks or pastures. The horses had unrestricted access to hay and straw, and concentrated feed was provided three times a day. Four of the horses were Polish Sport Horses and one was a pony. The horses were aged between 9 and 16 years. All the horses were in good health, with no visible signs of illness or injury, as confirmed by routine veterinary and physiotherapy check-ups. Their physical condition was good for recreational use and they exhibited no behavioral issues that would hinder participation in the study.

Each horse underwent basic dressage training and participated in regular recreational riding sessions lasting approximately six hours per week. The recreational horses were ridden by female riders aged between 19 and 23 years old with similar riding skills. The riding sessions included activities in walk, trot and canter. All sessions took place at the same time of day to control for diurnal variations in stress-related parameters.

The riding was performed outdoors in a designated training arena with a consistent sand-based surface. Efforts were made to ensure similar weather conditions for all sessions by scheduling them during the same season. Although temperature and humidity levels were not identical due to the variability inherent in outdoor settings, they were generally comparable and suitable for equine activity.

The recreational riding conducted during both the control and experimental phases was the same for each horse and followed the same schedule: walk—10 min, trot—16 min (with girth tightening in the second minute, changes in pace, stops, riding on curves, along a straight line and changes in direction), canter—8 min, and final walk—10 min.

In the experimental stage, each of the examined horses received a 45 min relaxation massage before the recreational ride. The massage was performed by a zoophysiotherapist who possessed the knowledge and skills to perform the massage. Each horse underwent the experiment twice, with a one-week interval between the experiments, following the same schedule on each test day to ensure consistency. The massage technique focused on working along the muscle fibers in the superficial layers of the muscles, concentrating on the muscles of the head, neck and back. The main techniques used during the procedure were stroking, rubbing and kneading. The primary goal of this form of massage was to stimulate circulation, which reduced muscle tension and thus relaxed the horse.

In the control and experimental stages, each horse was equipped with a heart-rate monitor (Polar s810i, Polar, Oy, Finland) consisting of a transmitter attached near the heart under the girth with an elastic strap and a receiver in the form of a watch attached to the saddle or the rider’s wrist. The transmitter picked up electrical impulses from the heart muscle and wirelessly transmitted them to the receiver, where the changing heart rate could be continuously monitored. The data were recorded in the device’s memory and then transferred to a computer for analysis using the Polar Trainer Equine Edition software. To facilitate the reading of data from the heart-rate monitor, the individual stages of working with the horses were marked using so-called phases (stages of working with a horse) markers.

Phases I and II in the trial without massage:

(1) Rider enters the stall;
(2) Tying the horse and beginning grooming;
(3) Removing the halter, putting on a bridle and leading to the riding arena;
(4) Walk;
(5) Canter;
(6) Dismounting from the horse;
(7) Cooling the legs;
(8) Horse in the stable;
(9) Removing the heart-rate monitor.

Phases I and II with massage:

(1) Rider enters the stall;
(2) Tying the horse and beginning grooming;
(3) Removing the halter, putting on a bridle and leading to the riding arena;
(4) Walk;
(5) Canter;
(6) Dismounting from the horse;
(7) Cooling the legs;
(8) Horse in the stable;
(9) Removing the heart-rate monitor.

Each horse underwent the research procedure twice, which included three stages:

- Zero stage (saliva sample collection to assess cortisol levels without recreational riding and massage at times corresponding to the hours of activities in the control and experimental stages);
- Control stage—without massage (recreational riding, heart-rate measurement, saliva sample collection to assess cortisol levels and assessment of horse behavior);
- Experimental stage—with massage (relaxation massage, recreational riding, heart-rate measurement, saliva sample collection to assess cortisol levels and assessment of horse behavior).

To determine the stress level related to the secretory activity of the adrenal cortex, the cortisol concentration in saliva was measured. Saliva samples were collected from the horses four times during the zero, control and experimental stages (Table 1). The sampling times for the zero, control and experimental stages were the same on each day of the study to eliminate the variability of cortisol level throughout the day.

To collect horse saliva, a Salivette^®^ swab was gently inserted into the horse’s mouth using forceps, and the sides of the cheeks, tongue and palate were wiped with it. After collecting the saliva, the swab was placed into a specially labeled tube and centrifuged for 15 min at 1500× *g*. The centrifuged saliva was transferred using a pipette into 1.5 mL Eppendorf tubes and it was then frozen at minus 17 °C until sent to the laboratory.

The cortisol concentration in saliva was determined using an ELISA test according to the manufacturer’s instructions, with some modifications to enhance the test’s sensitivity (Cortisol Enzyme Immunoassay Kit, Arbor Assays, MI, USA). Fifty microliters of standards and saliva samples or 75/50 µL of assay buffer (for non-specific binding sites and blanks, respectively), 25 µL of DetectX Cortisol Conjugate and DetectX Cortisol Antibody up to 100 µL were added into individual wells of the plate coated with goat anti-mouse IgG antibody. Each sample was applied to the plate in duplicate as technical replicates. The plate was incubated in the dark at 24 °C for one hour while shaking at 365 rpm using an ELMI Digital Thermostatic Shaker DTS-2 (ELMI, Riga, Latvia). Excess unbound antibodies were then washed off four times with 300 µL of Wash Buffer solution (BioTek™ ELx50 Microplate Strip Washer, BioTek, Winooski, VT, USA). Subsequently, 100 µL of TMB Substrate was added to the wells and these were incubated in the dark at 24 °C for 25 min. The reaction was stopped by adding 50 µL of Stop Solution, and the color was measured using an automated microplate spectrophotometer (BioTek Synergy 4 Hybrid Microplate Reader, BioTek) at a wavelength of 450 nm. The results were calculated using standard curves generated in each assay. The cortisol concentration is presented in pg/mL.

From the moment the rider entered the horse’s stall in both the control and experimental stages until the end of the ride and the return of the horse to the stall, the horse’s behavior was recorded. This allowed for precise observation of the number and types of conflict and relaxation behaviors. The duration of individual behaviors was not recorded; instead, a quantitative method was applied. Each behavior was noted, regardless of its duration, meaning that even brief occurrences were documented. There was no minimum time requirement for a behavior to be registered. Moreover, this approach was consistently applied across all behavior types, ensuring uniformity in data collection. This method allowed for a comprehensive analysis of both fleeting and prolonged behaviors. The recordings of the horses were analyzed by an independent behaviorist.

Conflict behaviors were categorized by type, and included ear pinning, head tossing, tail swishing, head raising, pawing, disobedience, wriggling, pulling on the reins, backing up, mouth opening, teeth grinding, biting and other, i.e., resistance to bridling and spooking. Relaxation behaviors recorded during the massage were also categorized by type, and included flattened ears, half-closed eyes, relaxed lower lip, sighing, yawning, snorting, defecation/urination, chewing, drooling and other, i.e., moving away from touch, leaning into touch.

The general outline of the research procedure, to which all subjects were subjected, was as follows:

1. Collection of the first cortisol sample approximately one hour before the ride and 10 min before the massage in the control and experimental stages;
2. Application of the heart-rate monitor approximately five minutes before the recreational ride and massage in the control and experimental stages;
3. Massage procedure in the experimental stage;
4. Collection of the second cortisol sample approximately 15 min before the start of horse preparation for the ride and after the massage in the control and experimental stages;
5. Grooming of the horse in the control and experimental stages;
6. Recreational riding in the control and experimental stages;
7. Cooling the horse’s legs after the ride in the control and experimental stages;
8. Unsaddling the horse in the control and experimental stages;
9. Returning the horse to the stall in the control and experimental stages;
10. Removal of the heart-rate monitor in the control and experimental stages;
11. Collection of the third cortisol sample approximately 15 min after the end of the ride in the control and experimental stages;
12. Collection of the fourth cortisol sample approximately 60 min after the end of the ride in the control and experimental stages.

All statistical calculations were performed using SAS 9.4. statistical package. The concentration of cortisol and the heart rate were log-normalized and analyzed using the GLIMMIX procedure, with an animal as a random effect accounting for the repeatability of measures. The sampling time, the order of the ride and the massage (performed or not) were main effects in cortisol analysis. For HR, the order of the ride, phase of the ride and the massage (performed or not) were used as main effects in the model. The occurrences of conflict/pain behaviors were totaled and their occurrence per minute was analyzed with the Friedman test. The occurrences of conflict/pain behaviors were totaled and their occurrence per minute was analyzed.

## 3. Results

### 3.1. Analysis of Salivary Cortisol Levels in the Horse Group

**Figure 1:**
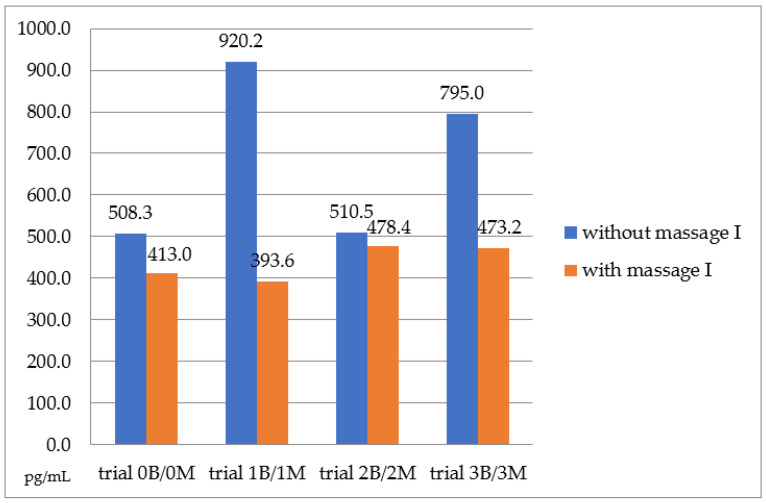
The average cortisol concentration from saliva (pg/mL) in all horses in the sample without massage I and after massage I.

Massage and the order of riding (first or second) significantly influenced cortisol levels, while the time of sample collection did not. Comparing the results of cortisol concentration between the sample without massage I and after massage I (Figure 1), a significant difference in hormone levels was observed in each trial. The most notable differences were found in trial 1B/1M, where cortisol decreased by 526.6 pg/mL after the massage and in trial 3B/3M, where cortisol dropped by 321.8 pg/mL. In the remaining trials, the decrease in cortisol concentration was also evident, although the differences were smaller.

### 3.2. Analysis of Conflict Behavior in the Horse Group

A statistically significant calming effect of massage on the total number of conflict behaviors was observed across different observation stages (before, during and after riding) (*p* = 0.0358). An analysis of the total number of conflict behaviors during the preparation phase of the horses revealed a significant difference between pre-massage and post-massage trials in the occurrence of undesirable behavior. Head tossing and ear pinning decreased after the massage. The most notable reduction was observed in nervous tail switching, which decreased 31 times after the massage. Other behaviors, such as biting and teeth grinding, were not exhibited during the preparation phase if the horse had received a massage.

The number of conflict behaviors observed during riding was considerably higher than during the preparation phase. Tail switching was the most common behavior, occurring 121 times in the pre-massage I trial and 143 times in the pre-massage II trial. After massage treatments, the number of conflict behaviors decreased, with some behaviors completely disappearing. Tail switching was observed 36 times less after the first massage and 53 times less after the second massage. Additionally, ear pinning and mouth opening during riding occurred significantly less in massaged horses compared to those that were not massaged. Behaviors that were not observed in horses after massage included head tossing, rein pulling and backing up.

In the third phase of the study, after the ride, conflict behavior was minimal. In the pre-massage I trial, horses most frequently exhibited head tossing (five times) and tail switching (three times). After the massage, only head tossing (one time) and hoof striking (four times) were observed, with other behaviors absent. In the pre-massage II trial, tail switching was most common (16 times), followed by ear pinning (5 times). After the second massage, only tail switching was observed, with no other behavior present.

Overall, the data indicated that the number of undesirable behaviors decreased after each massage compared with the non-massaged condition. Additionally, during the second massage, it was noted that horses began to habituate to the tactile stimuli used during the procedure.

In the present study, highly significant differences were observed between the average number of behaviors and the stage of work (Table 2). The highest number of conflict behaviors occurred during the riding stage, averaging 32.45 times, suggesting that this stage may be the most stressful for horses.

The study also found that massage had an effect on reducing the number of conflict behaviors in the studied horse population, supporting the hypothesis that massage relaxes and calms these animals (Table 3).

### 3.3. Analysis of Relaxation Behavior During Massage in the Horse Group

A significant calming effect of massage on the total number of conflict behaviors during the observation stages was noted. During the massage sessions, the horses displayed relaxation behavior indicative of body relaxation and ease (Table 4). The most frequently observed behavior was a relaxed lower lip, which occurred 6 times after the first massage and 17 times after the second massage. Another notable relaxation behavior was the half-closed eyes, which made the horses appear as if they were dozing off at times. The average for this behavior was 9.4 occurrences after the first massage and 13.6 after the second massage. Chewing was also an important behavior to note. After the first massage, the horses chewed an average of 8.4 times, while after the second massage, this increased to an average of 10.6 times. It is commonly understood in equestrian circles that horses only chew when they are relaxed. Chewing is also a sign that the horse comprehends and accepts the situation. Other significant signs of relaxation included deep sighs or exhalations and ears that were relaxed and laid to the sides. It is worth noting that the frequency of these desirable behaviors increased after the second massage. A significant difference in flattened ears to the side was recorded between the first and second massage session (*p* = 0.0253).

### 3.4. Analysis of Heart-Rate Values in the Horse Group

Massage had a nearly significant effect on heart rate (*p* = 0.057), whereas the order of riding did not. Statistical analysis also showed highly significant differences between the average heart rate and the phase number in trials without massage. In phase 7, a significant decrease in heart rate was observed, attributed to the relaxing activity of cooling the legs after riding, with a reduction of 23.8 bpm. The heart monitor was removed after the horses entered their stalls (phase 9), showing an average heart rate of 49.00 bpm, which was only slightly higher than the initial rate (Table 5).

Statistical analysis revealed highly significant differences between the average heart rate and the phase number in trials. At the beginning of grooming (phase 2), the horses’ average heart rate was 45.90 bpm. When the horse started walking (phase 4), the calculated average heart rate was 51.61 bpm, while in the gallop phase (phase 5), the heart rate increased to 85.80 bpm. In phase 7, the average heart rate recorded during leg cooling was 60.00 bpm, a decrease of 25.20 bpm compared to the gallop. The heart monitor was removed after the horse was returned to the stall (phase 9), with an average heart rate of 50.20 bpm, only slightly higher than the resting rate (Table 6). A highly significant decrease in heart rate after riding, compared to the heart rate during dismounting, was observed during leg washing (*p* < 0.0001).

## 4. Discussion

The current study found that the horseback riding stage caused more conflict behavior in horses. Different studies investigating the effects of rein tension on horse behavior and physiology found a significant relationship between rein tension and increased heart rate, elevated salivary cortisol and conflict behavior, indicating that riders can induce discomfort in horses [21].

Długosz et al. [22] aimed to assess salivary cortisol levels in recreational horses based on their type of activity. The experiment involved 68 horses categorized by recreational activity, type, age and gender. Saliva samples were collected from each horse three times daily. The analysis revealed that both recreational activity and age significantly influenced salivary cortisol levels. However, no significant differences in daily cortisol levels were found between different types of horses or between mares and geldings. Notably, cortisol concentrations in saliva after recreational riding were significantly higher compared with other activity levels like lunging, schooling in and out or hippotherapy. Across all groups, physical activity consistently led to an increase in cortisol levels. In the current study, the highest number of conflict behaviors was observed during the riding stage, suggesting that this phase may be the most stressful for horses.

In the current study, significant differences were observed between the average number of behaviors and the stage of work. The highest number of conflict behaviors occurred during the horseback riding phase, suggesting that this stage may be the most stressful for horses. Statistical analysis revealed highly significant differences between the average heart rate and the phase number in trials. After the riding stage, during the leg cooling, a significant decrease in heart rate was noted and showed an average heart rate that was only slightly higher than the initial rate. The heart-rate monitor was removed after the horses returned to their stalls. The current results demonstrate a reduction in cortisol levels following massage, which acted as a relaxing factor and helped balance the stress associated with activity. In contrast, the study by Kang et al. [13] showed that cortisol levels were higher in a non-exercising group compared with those engaged in physical activity, which is the opposite of the current findings.

The current study highlights the effectiveness of massage as a non-invasive method of reducing stress in recreational horses. Similar to the work by Birt et al. [5], the authors of the current study observed that massage induced a state of relaxation in the horses, demonstrated by a decrease in heart rate and the occurrence of relaxation-related behavior. McBride et al. [23] and Kędzierski et al. [24] also reported physiological and behavioral benefits of massage, including reduced cortisol levels and fewer stress-related behaviors, which is consistent with the current results.

Moreover, while previous studies have explored the effects of massage frequency [25] and its impact on performance [26], the current research emphasizes its immediate calming effects on conflict behavior and cortisol level reduction. The observed behavioral responses, such as relaxed postures and reduced tension, underline massage’s role in enhancing horse well-being.

In contrast to studies involving water cooling [27], which primarily target physiological relaxation post-exercise, massage provides both physical and psychological benefits. This dual impact not only reduces stress but also fosters a deeper sense of calm, suggesting that massage could be an integral part of routine care for horses. These findings contribute to the growing evidence supporting massage as a practical tool for improving equine welfare.

Massage positively affected the well-being of the recreational horses by lowering levels of cortisol, known as the stress hormone. The horses accepted the massage treatments, demonstrating this through relaxing behavior such as chewing, half-closing their eyes, snorting, laying their ears to the sides and relaxing their lower lip. This study showed that massage reduced the number of conflict behaviors in the studied population of horses, proving that massage relaxed and calmed these animals. After the massage treatments, the horses exhibited fewer conflict behaviors, and some of these behaviors disappeared entirely.

## 5. Conclusions

The research conducted in this study aimed to demonstrate the impact of massage treatments on selected physiological and behavioral parameters in horses These parameters illustrated the stress experienced by recreationally used animals. Horses subjected to massage showed a significant decrease in salivary cortisol levels and a reduction in conflict behavior. The research conducted on the influence of massage on the well-being of the horses indicated that this treatment had a beneficial effect on the horse’s body, reducing stress by relieving the pain of working muscles, as well as promoting relaxation and calmness. Evidence for this included the observed relaxing behavior that the horses displayed during the massage treatments. Additionally, it was found that cooling the horses’ legs with water led to a decrease in heart rate, which promoted relaxation. The obtained results have practical applications for horse owners and caretakers during animal use and in further studies on the impact of massage on horse well-being.

## Figures and Tables

**Table 1:** Cortisol sampling times.

Zero Stage	Control Stage, Without Massage	Experimental Stage, With Massage
10:50 cortisol 00	10:50 cortisol 0B	10:50 cortisol 0M
-	-	11:00 massage
11:45 cortisol 01	11:45 cortisol 1B	11:45 cortisol 1M
-	12:00 grooming	12:00 grooming
-	12:15–13:15 recreational riding	12:15–13:15 recreational riding
13:30 cortisol 02	13:30 cortisol 2B	13:30 cortisol 2M
14:15 cortisol 03	14:15 cortisol 3B	14:15 cortisol 3M

B—without massage; M—with massage.

**Table 2:** The number of conflict behaviors, considering the stages before riding, during riding and after riding in the studied horse population.

	N	Mean	SD
Before riding	20	11.55 ^B^	7.06
During riding	20	32.45 ^A^	20.58
After riding	20	1.90 ^C^	3.07
Overall	60	15.30	17.91

N—total number of samples in a study; A,B,C—values within columns for the same trait marked with different letters differ statistically; uppercase letters indicate significance at α = 0.01.

**Table 3:** Conflict behavior in horses considering massage treatments.

	N	Mean	SD
Without massage	30	19.86 ^a^	21.77
With massage	30	10.73 ^b^	11.64
Overall	60	15.30	17.91

N—total number of samples in a study; a,b—values within columns for the same trait marked with different letters differ statistically; lowercase letters indicate significance at α = 0.05.

**Table 4:** Total number of relaxation behaviors observed during massage I and massage II.

Horse Number	Relaxation Behavior During Massage I/Massage II								
	Half-Closed Eyes	Relaxed Lower Lip	Snorting	Flattened Ears	Defecation/Urination	Sighing	Chewing	Yawning	Drooling
1.	1/2	3/6	4/9	4/10	1/1	1/4	5/11	0/0	0/0
2.	6/20	7/21	2/6	2/11	0/0	0/2	10/16	0/2	0/0
3.	10/16	17/21	1/2	7/10	0/0	2/5	10/8	1/2	0/4
4.	15/15	11/24	4/2	7/12	0/1	1/0	7/10	2/4	0/0
5.	15/15	15/13	2/0	5/12	0/1	4/2	10/8	0/0	0/0

**Table 5:** Heart rate considering the phase number in trials without massage.

Phase	N	Mean	SD
1	10	44.30 ^E,F^	2.98
2	10	50.10 ^D,E^	8.36
3	10	42.60 ^F^	3.95
4	10	44.20 ^E,F^	4.47
5	10	84.60 ^B^	3.69
6	10	99.40 ^A^	5.99
7	10	60.80 ^C^	5.79
8	10	54.80 ^D^	3.74
9	10	49.00 ^D,E^	4.22
Overall	90	58.87	19.54

N—total number of samples in a study; A,B,C,D,E,F—values within columns for the same trait marked with different letters differ statistically; uppercase letters indicate significance at α = 0.01.

**Table 6:** Heart rate considering the phase number in trials after massage.

Phase	N	Mean	SD
1	10	49.32 ^A,B^	3.27
2	10	49.50 ^A^	4.67
3	10	43.72 ^A^	2.45
4	10	51.61 ^B^	13.60
5	10	85.80 ^D^	8.93
6	10	88.80 ^D^	12.86
7	10	60.00 ^C^	2.54
8	10	55.90 ^B,C^	4.09
9	10	50.20 ^A,B^	1.62
Overall	90	57.11	16.30

N—total number of samples in a study; A,B,C,D—values within columns for the same trait marked with different letters differ statistically; uppercase letters indicate significance at α = 0.01.

## Data Availability

All data generated or analyzed during this study are included in this published article.
